# A High-Density Genetic Map Identifies a Novel Major QTL for Boron Efficiency in Oilseed Rape (*Brassica napus* L.)

**DOI:** 10.1371/journal.pone.0112089

**Published:** 2014-11-06

**Authors:** Didi Zhang, Yingpeng Hua, Xiaohua Wang, Hua Zhao, Lei Shi, Fangsen Xu

**Affiliations:** National Key Laboratory of Crop Genetic Improvement, and Microelement Research Centre, Huazhong Agricultural University, Wuhan, China; The University of Western Australia, Australia

## Abstract

Low boron (B) seriously limits the growth of oilseed rape (*Brassica napus* L.), a high B demand species that is sensitive to low B conditions. Significant genotypic variations in response to B deficiency have been observed among *B. napus* cultivars. To reveal the genetic basis for B efficiency in *B. napus*, quantitative trait loci (QTLs) for the plant growth traits, B uptake traits and the B efficiency coefficient (BEC) were analyzed using a doubled haploid (DH) population derived from a cross between a B-efficient parent, Qingyou 10, and a B-inefficient parent, Westar 10. A high-density genetic map was constructed based on single nucleotide polymorphisms (SNPs) assayed using *Brassica* 60 K Infinium BeadChip Array, simple sequence repeats (SSRs) and amplified fragment length polymorphisms (AFLPs). The linkage map covered a total length of 2139.5 cM, with 19 linkage groups (LGs) and an average distance of 1.6 cM between adjacent markers. Based on hydroponic evaluation of six B efficiency traits measured in three separate repeated trials, a total of 52 QTLs were identified, accounting for 6.14–46.27% of the phenotypic variation. A major QTL for BEC, *qBEC-A3a*, was co-located on A3 with other QTLs for plant growth and B uptake traits under low B stress. Using a subset of substitution lines, *qBEC-A3a* was validated and narrowed down to the interval between CNU384 and BnGMS436. The results of this study provide a novel major locus located on A3 for B efficiency in *B. napus* that will be suitable for fine mapping and marker-assisted selection breeding for B efficiency in *B. napus*.

## Introduction

Boron (B) is one of the essential microelements for plant growth and development [1]. The main function of B is in the formation and structural integrity of the plant cell wall, where it cross-links rhamnogalacturonan II (RG-II) [2]–[5]. Unfortunately, low B soils are present in many regions of the world, with B deficiency reported in over 80 countries and for 132 crops [6]. Growing tissues exhibit the first symptoms of B deficiency, including inhibition of root elongation and leaf expansion and reduction of fertility, which ultimately cause declines in crop yield and quality [7].

Oilseed rape (*Brassica napus* L.) is one of the major oilseed crops in the world, providing vegetable oil for human consumption, stock feed for animals and bio-fuel for industry [8]. Among crop species, *B. napus* has a high requirement for B and is sensitive to B deficiency [9]. The typical symptom of B deficiency in *B. napus*, “flowering without seed setting”, was first reported in the 1970s in China [10]. Over the past 20 years, the application of B fertilizers for crop production has been a standard measure to solve the problem of B deficiency in soils with a hot water soluble B (HWB) concentration below 0.5 mg kg^−1^ [10]. However, the narrow range between B deficiency (<0.5 mg kg^−1^ HWB) and B toxicity (>1.0 mg kg^−1^ HWB) makes the application of B fertilizers risky. Boron can be toxic to some crops at soil B levels only slightly above those required for normal growth in other crops. Moreover, borate rock is a limited and non-renewable mineral resource. Therefore, breeding new B-efficient varieties represents an important and practical method to improve the production of *B. napus* in B-deficient soils.

The uptake and transport of B are regulated by a series of genes in plants, particularly under low B conditions. In *Arabidopsis*, the influx of B from B-deficient soil into root cells is accomplished by *AtNIP5;1*, a boric acid channel, and the efflux of B out of the cells toward the xylem is controlled by *AtBOR1*, a B transporter for xylem loading [11]–[13]. The overexpression of *AtBOR1* or *AtNIP5;1* can improve B deficiency tolerance and seed yield in *Arabidopsis* [14]–[15]. In recent years, B transporters have been isolated in several crops, including rice (*Oryza sativa* L.) [16], wheat (*Triticum aestivum* L.) [17] and barley (*Hordeum vulgare* L.) [18]. In *B. napus*, six B transport genes, *BnBOR1*s, homologous to *AtBOR1* have been cloned. These genes show the same or different gene structures and expression profiles as *AtBOR1* [19].

Following the definition of Graham [20], B efficiency refers to the ability of a genotype to grow well and produce a high yield in soils with a given HWB level that would be deficient for standard cultivars. Significant genotypic differences in response to B deficiency exist among *B. napus* cultivars [21]–[25]. Quantitative trait loci (QTLs) for B efficiency in *B. napus* have been analyzed using several populations [26]–[28]. One major QTL for B efficiency, *BE1*, as well as another three QTLs were detected in a F_2_ population derived from a cross between the B-efficient parent Qingyou 10 and the B-inefficient parent Bakow [26]. Zhao et al, [27] used these two parents to construct a doubled haploid (DH) population and analyzed the QTLs for seed yield and yield-related traits. The QTLs for seed yield and the B efficiency coefficient (BEC) located on the A2 linkage group were equivalent to the *BE1* region. However, the phenotypic variations in the QTLs detected in the DH population were lower than those detected in the F_2_ population. Another locus for B utilization efficiency, *BnBE2*, was detected in an advanced backcross population by bulk segregant analysis (BSA) [28]. However, these previous QTLs mapping studies were based on genetic maps with the low marker densities. Recently, a 60,000 (60 K) single nucleotide polymorphism (SNP) Infinium genotyping array for *B. napus* was produced by the international *Brassica* SNP consortium in cooperation with Illumina Inc. San Diego, CA, USA [29]–[30], which opens the way for efficient and low-cost construction of a high-density genetic map of *B. napus*. High-density genetic maps can improve the precision of QTLs localization and the accuracy of effect for the detected QTLs [31].

In this study, a newly developed *B. napus* DH population, named the QW DH population, was genotyped using *Brassica* 60 K Infinium SNP array, simple sequence repeats (SSRs) and amplified fragment length polymorphisms (AFLPs) markers to construct a high-density genetic map. The map was then used to detect QTLs for plant growth, B uptake and BEC using trait data from three hydroponic trials with the population grown under high and low B conditions. The results will provide major QTLs suitable for fine mapping and the physically adjacent markers for breeding B efficiency in *B. napus*.

## Materials and Methods

### Plant materials and hydroponics

A DH population containing 70 lines derived from a cross between the B-efficient parent Qingyou 10 and the B-inefficient parent Westar 10, which is more sensitive to B deficiency than the B-inefficient parent Bakow [32], named the QW DH population, was used to construct a high-density genetic map and identify QTLs associated with plant growth, B uptake and BEC.

Fifty seeds of each line of the QW DH population were randomly selected and weighed. After incubation at 4°C for 12 h, the seeds were sowed on gauze, fixed on a black plastic tray filled with deionized water, and germinated for 6 days. Uniform seedlings were transplanted to plastic containers containing nutrient solution, and the original primary root length (PRL_0d_) of every plant was measured. The base nutrition solution contained 1.0 mM KH_2_PO_4_, 5.0 mM KNO_3_, 5.0 mM Ca(NO_3_)_2_•4H_2_O, 2.0 mM MgSO_4_•7H_2_O, 0.05 mM EDTA-Fe, 9 µM MnCl_2_•4H_2_O, 0.8 µM ZnSO_4_•7H_2_O, 0.3 µM CuSO_4_•5H_2_O, and 0.1 µM Na_2_MoO_4_•2H_2_O, This solution was then supplied with a high B (HB) level of 25 µM H_3_BO_3_ or a low B (LB) level of 0.25 µM H_3_BO_3_. The parental lines were cultivated in each container as controls. The experiment was conducted as a randomized complete block design with three replicates. The initial nutrient solution was a one-quarter strength solution that was replaced every 5 days, followed by one-half strength and finally full-strength. All lines were grown for 21 days in an illuminated culture room at 22°C under a 14 h light/10 h dark cycle. The photosynthetic photon flux density and relative humidity were 300–320 µmol/m^2^/s and 60–75%, respectively. The hydroponics experiment was independently replicated three times, in June of 2010, October of 2011 and July of 2012, respectively.

### Trait measurement

Upon harvesting, the primary root length (PRL_21d_) was measured, and the increment of primary root length (IPRL = PRL_21d_-PRL_0d_) was calculated. The shoot and root samples were separated and dried at 105°C for 30 min and then to a constant weight about 48 h at 65°C. The dried samples were ground to fine powder, 0.1000 g and 0.0450 g of which were weight and 10 mL and 6 mL of 1 M HCl were then added to extract B in the shoot and root samples, respectively [33]. The B concentrations were measured by inductively coupled plasma atomic emission spectroscopy (ICP-AES) using an IRIS Advantage instrument (Thermo Electron, USA). Boron accumulation in the shoot and root was calculated as the B concentration × dry weight, and the ratio of the plant dry weight in LB to that in HB was defined as the BEC.

### SNP marker analysis and linkage analysis

Two parental lines and 70 QW DH lines were genotyped using the *Brassica* 60 K SNP BeadChip Array developed by the international *Brassica* SNP consortium in cooperation with Illumina Inc. San Diego, CA, USA. The array hybridization, including DNA sample preparation, hybridization to the BeadChip, washing, primer extension and staining were performed according to the work flow described in the Infinium HD Assay Ultra manual provided by array manufacturer (Illumina, San Diego, CA). Imaging of the arrays was performed using an Illumina HiSCAN scanner after BeadChip washing and coating. Calling SNP genotype data using the BeadStudio genotyping software generally produced three clear clusters: AA homozygote, BB homozygote and AB heterozygote. Of the 52,157 SNPs in the array, those SNPs with an AA or BB frequency equal to zero, missing data >0.05 or those SNPs that did not show three clearly defined clusters (AA, BB and AB) were excluded. Thus, 11,080 SNPs were selected according to the SNP genotype data analysis. Further, those molecular markers with identical genotypes across the QW DH population were classified into a bin by Perl language. Finally, the selected 11,080 SNPs were grouped into 1,346 SNP bins, which included 1 to 1,090 SNP markers in each bin. Primer sequences of SSR markers were obtained from various public sources: UK prefixed by OL and Na (http://www.brasscia.bbsrc.ac.uk/BrassicaDB), Australia prefixed by sA (http://www.hornbill.cspp.latrobe.edu.au), Canada prefixed by sR and sN (http://www.brassica.agr.gc.ca/index_e.shtml), Japan prefixed by BRMS [34], France prefixed by BRAS, CB and MR [35], BnGMS [36] and BoGMS [37], private communications prefixed by CNU and niab and a total of 171 *Brassica rapa* BAC sequence and/or BAC-end sequence primers [38].

AFLP markers were analysed following Vos et al. [39] using fluorescently labeled *Sac*I and *Mse*I primers with three selective nucleotides, as described by Zhao et al. [40]. AFLP markers were named using a code for each *Sac*I and *Mse*I primer followed by the molecular weight.

Linkage analysis was performed using the software program JoinMap 4.0 [41], applying the mapping function of Kosambi [42] with a minimum LOD score of 4.0 and a maximum recombination fraction of 0.4. A SSR framework map was firstly constructed based on the order of the SSR markers in a previous study [27], and then SNP-bin and AFLP markers were added to construct the high-density genetic map.

### QTL analysis

QTL analysis was performed on the QW DH population with the composite interval mapping (CIM) model [43] using the WinQTL Cartographer 2.5 software [44]. The threshold LOD score for each trait was determined by performing a 1000-permutation test at a significant level of *P*<0.05 [45]. All QTLs identified were named using the initial letter ‘*q*’, followed by the abbreviation of the trait and B level, and then suffixed with the corresponding linkage group. If there was more than one QTL for the same trait detected in the same linkage group, a serial letter was added to the end of the name of the QTL. For example, *qIPRLHB-A8a* indicates that the first QTL for IPRL was detected at the HB in the A8 linkage group.

### Phenotype of substitution lines

A BC_4_F_1_ population was constructed by backcross using Westar 10 as the recurrent parent and Qingyou 10 as the donor parent. From the BC_4_F_1_ population, one plant heterozygous for the target QTL, *qBEC-A3a*, and homozygous for other QTLs loci with Westar 10 was selected to produce BC_4_F_2_. Using eight linked SSR markers (CNU384, BnGMS436, BnGMS20, BoGMS0843, CNU098, BOGMS1117a, B043L02-1, FITO285) at the confidence interval of *qBEC-A3a*, six substitution lines were selected from the BC_4_F_2_ population, and then twenty-four BC_4_F_2;3_ plants for each substitution line were cultivated in nutrient solutions with high B (25 µM H_3_BO_3_) or low B (0.25 µM H_3_BO_3_). The average BEC and SDW at LB of the twenty-four BC_4_F_2:3_ plants were used to evaluate the BEC and SDW at LB of the substitution line.

### Statistical analysis

In total, six traits, including three plant growth traits, two B uptake traits and the BEC, were used for normality, variance and QTL analyses. Pearson's correlation analysis was performed to examine the phenotypic association. The frequency distribution of the QW DH population for all tested traits was established using SPSS 17.0 software (SPSS, Chicago, IL, USA). The broad-sense heritability estimate (*h^2^*) was calculated for each trait as described by Shi et al. [46].

## Results

### High-density SNP map construction

A high-density linkage map for the QW DH population was constructed using SSR, SNP-bin and AFLP markers, producing a map containing 19 linkage groups representing the *B. napus* chromosomes of the A genome (A1-A10) and the C genome (C1-C9). The map included 936 SNP-bins, 343 SSR and 119 AFLP markers and had a total length of 2,139.5 cM, with an average distance of 1.6 cM between adjacent markers, covering 1,070.5 and 1,068.0 cM for the A and C genomes, respectively. The number of marker bins in each group ranged from 36 to 120, and the length of each group ranged from 40.5 to 209.4 cM (Table 1, File S1).

**Table 1:** Summary of the total number of markers, map length and average distance between adjacent loci of the *Brassica napus* high-density linkage map based on the QW DH population derived from a cross between the B-efficient parent Qingyou 10 and the B-inefficient parent Westar 10.

Linkage group	Total number of markers (SNP-bin+SSR+AFLP)	Map length (cM)	Average distance between adjacent loci (cM)
A1	90 (58+25+7)	100.8	1.1
A2	79 (59+17+3)	70.1	0. 9
A3	120 (80+34+6)	180.7	1.5
A4	72 (54+13+5)	94.0	1.3
A5	96 (63+23+10)	135.2	1.4
A6	65 (49+8+8)	129.9	2.0
A7	73 (53+11+9)	80.6	1.1
A8	63 (44+14+5)	101.0	1.6
A9	65 (36+25+4)	106.9	1.6
A10	55 (43+9+3)	71.3	1.3
C1	65 (34+20+11)	110.4	1.7
C2	47 (19+19+9)	40.5	0.7
C3	106 (74+27+5)	160.1	1.5
C4	73 (48+22+3)	100.3	1.4
C5	36 (25+11+0)	73.5	2.0
C6	42 (34+4+4)	105.0	2.5
C7	81 (53+17+9)	140.4	1.7
C8	83 (54+22+7)	129.4	1. 6
C9	87 (56+20+11)	209.4	2.4
A genome	778 (539+179+60)	1070.5	1.4
C genome	620 (397+164+59)	1069.0	1.7
Total	1398 (936+343+119)	2139.5	1.6

### Phenotypic variation of the tested traits in QW DH population

**Figure 1:**
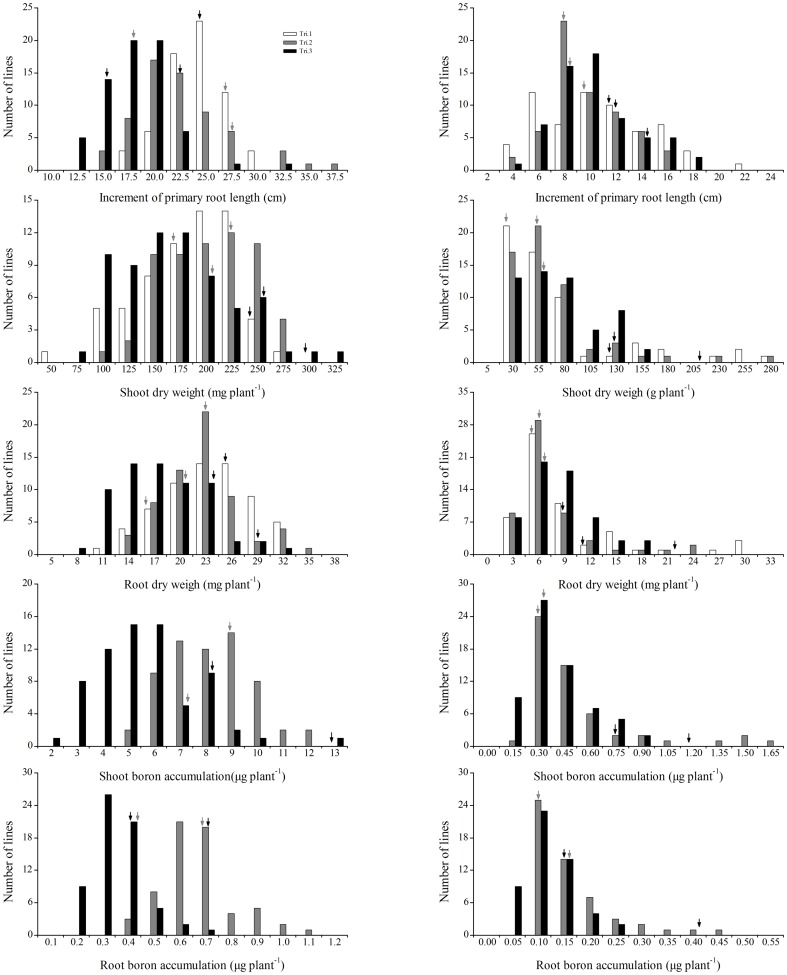
Frequency distributions of plant growth and B uptake traits at high B (left) and low B (right) in the QW DH population derived from a cross between the B-efficient parent Qingyou 10 and the B-inefficient parent Westar 10. The black arrows indicate Qingyou 10, and the gray arrows indicate Westar 10.

The mean, range and heritability (*h^2^*) values for the six tested traits in the QW DH population and their parental lines are summarized in Table 2. The values of shoot dry weight (SDW), root dry weight (RDW), shoot B accumulation (SBA) and root B accumulation (RBA) under both B levels and the BEC of the B-efficient parental line Qingyou 10 were higher than those of the B-inefficient parental line Westar 10, especially at LB (Table 2). For the increment of primary root length (IPRL), Westar 10 was longer than Qingyou 10 under the HB condition; however, the opposite trend was observed at LB (Table 2). The tested traits exhibited wide ranges of variation in the QW DH population at both B levels (Table 2, Fig. 1). In general, the coefficient of variations (CV%) for the plant growth and B uptake traits were higher at LB than at HB, implying that significant genotypic variation in response to B deficiency existed in the QW DH population. Under LB, the plant growth traits and BEC had higher *h^2^* than the B uptake traits, indicating a more stable heritability and fewer environmental effects on plant growth and BEC (Table 2). The frequency distributions of plant growth and B uptake traits showed continuous and transgressive segregation in the QW DH population exposed to both B levels (Fig. 1), suggesting that multiple genes are involved.

**Table 2:** Mean, range and broad-sense heritability (*h^2^*) of tested traits under high and low B in the QW DH population derived from a cross between Qingyou 10 and Westar 10 and their parental lines.

Trait	B level	Trial	Parental lines		QW DH lines			*h^2^*
			Qingyou 10	Westar 10	Mean	Range	CV%	
IPRL (cm)	HB	1	22.82±0.71	25.28±1.37	23.04	17.12–28.29	11.90	0.19
		2	21.30±0.49	25.34±1.87	21.29	13.10–36.16	21.93	
		3	15.73±0.92	16.29±4.79	16.99	10.09–31.02	19.75	
	LB	1	11.61±1.26	9.26±1.31	9.68	2.23–21.62	42.84	0.84
		2	10.46±1.14	7.04±0.43	8.62	2.33–14.96	33.50	
		3	11.95±1.80	7.36±1.66	9.23	2.97–17.23	34.52	
SDW (mg plant^−1^)	HB	1	245.17±13.13	157.87±5.94	171.9	48.77–271.58	26.99	0.41
		2	289.23±29.94	217.26±46.89	189.6	98.58–267.18	22.25	
		3	205.48±47.78	177.87±12.00	156.4	72.78–316.43	35.67	
	LB	1	118.43±12.41	28.68±1.66	63.78	13.77–255.89	94.58	0.82
		2	123.04±23.68	31.24±2.10	56.39	9.40–256.42	83.25	
		3	195.99±36.35	46.00±6.56	62.88	10.07–146.66	57.35	
RDW (mg plant^−1^)	HB	1	24.56±2.98	17.64±3.06	21.75	10.90–30.64	22.75	0.46
		2	27.24±3.67	19.32±1.76	21.06	11.93–33.28	20.86	
		3	22.18±2.48	19.08±2.68	16.13	7.583–30.18	32.03	
	LB	1	12.39±2.24	4.02±0.37	7.81	0.84–29.69	88.71	0.85
		2	13.47±1.04	3.61±0.26	6.31	1.48–22.67	73.49	
		3	16.56±4.66	5.97±0.46	6.78	1.05–16.11	54.05	
SBA (µg plant^−1^)	HB	2	12.56±0.76	8.10±1.87	7.65	4.55–11.68	20.71	0.30
		3	7.19±1.87	6.30±0.37	5.15	1.59–12.08	37.52	
	LB	2	0.72±0.16	0.27±0.03	0.43	0.15–1.64	76.62	0.65
		3	1.13±0.26	0.25±0.04	0.32	0.09–0.84	57.29	
RBA (µg plant^−1^)	HB	2	0.69±0.08	0.55±0.03	0.62	0.34–1.01	23.05	0.43
		3	0.43±0.05	0.38±0.05	0.31	0.15–0.68	33.31	
	LB	2	0.23±0.02	0.07±0.01	0.13	0.05–0.42	60.22	0.62
		3	0.29±0.08	0.12±0.01	0.09	0.03–0.22	48.89	
BEC		1	0.48±0.03	0.19±0.01	0.38	0.08–1.66	94.01	0.82
		2	0.42±0.02	0.15±0.02	0.31	0.07–1.52	82.80	
		3	0.85±0.15	0.27±0.04	0.40	0.06–1.14	63.10	

IPRL, increment of primary root length; SDW, shoot dry weight; RDW, root dry weight; SBA, shoot B accumulation; RBA, root B accumulation; BEC, B efficiency coefficient; HB, high B; LB, low B.

Significant positive correlations were observed between plant growth and B uptake traits at the two B levels, especially for SDW and SBA, RDW and RBA (Table 3). Interestingly, BEC showed significant positive correlations with plant growth and B uptake traits at LB; however, no significant correlation with those was found at HB (Table 3).

**Table 3:** Correlation coefficients between the tested traits under high (above diagonal) and low B (below diagonal) in the QW DH population derived from a cross between Qingyou 10 and Westar 10.

	IPRL	SDW	RDW	SBA	RBA	BEC
IPRL		0.29*	0.39**	0.28*	0.29*	ns
SDW	0.58**		0.83**	0.94**	0.73**	ns
RDW	0.58**	0.96**		0.79**	0.89**	ns
SBA	0.48**	0.91**	0.86**		0.72**	ns
RBA	0.59**	0.94**	0.97**	0.90**		ns
BEC	0.62**	0.85**	0.86**	0.85**	0.81**	

IPRL, increment of primary root length; SDW, shoot dry weight; RDW, root dry weight; SBA, shoot B accumulation; RBA, root B accumulation; BEC, B efficiency coefficient.

*P<0.05;

**P<0.01; ns, no significance.

### QTL for plant growth traits

A total of 36 QTLs were associated with plant growth, including 15 and 21 QTLs identified at HB and LB, respectively (Table 4). Each QTL accounted for 6.14–46.27% of the phenotypic variation, with 32 individually explaining more than 10% of the phenotypic variance. Favorable alleles for increasing plant growth were contributed by Qingyou 10 at 22 loci and by Westar 10 at 14 loci (Table 4). Furthermore, 15 QTLs were found for IPRL, 7 of which were detected at HB and 8 at LB. Among them, two constitutive loci for IPRL were mapped on A5 and A8, where *qIPRLHB-A5* and *qIPRLLB-A5* co-located on A5 and *qIPRLHB-A8a* and *qIPRLLB-A8* co-located on A8. Ten and eleven QTLs respectively controlling SDW and RDW were identified. Co-localization of the QTLs for SDW and RDW were found on A3, A4, A5, C3 and C9, implying that SDW and RDW are genetically closer, especially at LB. Three major QTLs, *qIPRLLB-A3* for IPRL, *qSDWLB-A3a* for SDW and *qRDWLB-A3a* for RDW, individually explaining 46.27, 40.22 and 43.93% of the phenotypic variation, co-located on the same region of A3 and were consistently detected in two or three trials at LB.

**Table 4:** QTLs detected for plant growth, B uptake traits and BEC under high and low B using the QW DH population derived from a cross between Qingyou 10 and Westar 10.

B level	Trait	LG*	QTL	Peak†	CI‡	LOD§	R^2^ (%)¶	Add.#	Tri.ξ
HB	IPRL	A5	*qIPRLHB-A5*	48.2	45.6–50.9	3.62	13.27	-	2
		A8	*qIPRLHB-A8a*	23.0	22.5–29.3	3.18	12.88	+	3
		A8	*qIPRLHB-A8b*	74.2	73.4–74.8	3.67	13.11	+	1
		A10	*qIPRLHB-A10*	10.5	10.1–15.4	4.86	19.99	-	3
		C4	*qIPRLHB-C4*	81.2	77.2–86.3	3.60	12.85	+	2
		C8	*qIPRLHB-C8*	10.5	9.0–12.8	5.18	20.84	-	2
		C9	*qIPRLHB-C9*	151.7	149.4–159.9	2.96	10.44	+	1
	SDW	C4	*qSDWHB-C4*	35.7	35.6–35.8	3.23	13.74	-	3
		C7	*qSDWHB-C7*	117.3	114.2–120.3	3.72	13.15	+	2
		C8	*qSDWHB-C8*	0.0	0.0–1.3	3.76	13.62	-	2
		C9	*qSDWHB-C9*	130.6	129.8–137.1	5.22	20.91	-	1
	RDW	C2	*qRDWHB-C2*	0.0	0.0–3.7	3.35	15.59	+	3
		C4	*qRDWHB-C4*	71.5	62.5–72.0	4.48	18.67	+	1, 2
		C7	*qRDWHB-C7*	128.2	121.4–128.9	4.39	18.50	+	2
		C9	*qRDWHB-C9*	130.6	129.8–132.3	4.02	15.32	-	1
	SBA	A1	*qSBAHB-A1*	3.8	2.0–4.6	4.19	16.09	-	3
		C1	*qSBAHB-C1*	9.7	8.8–14.4	3.72	15.39	-	2
	RBA	A8	*qRBAHB-A8*	3.0	0.0–14.8	3.43	16.79	+	3
		C7	*qRBAHB-C7*	128.6	128.2–128.9	4.91	22.86	+	2
LB	IPRL	A2	*qIPRLLB-A2*	68.1	67.2–68.6	3.61	8.42	+	1
		A3	*qIPRLLB-A3*	86.5	81.8–87.6	13.49	46.27	+	1, 2
		A5	*qIPRLLB-A5*	42.9	38.6–48.2	7.21	19.34	+	1
		A6	*qIPRLLB-A6*	80.7	79.4–89.2	5.70	14.14	+	2
		A8	*qIPRLLB-A8*	22.5	14.4–28.5	5.58	14.50	+	3
		A9	*qIPRLLB-A9*	34.3	31.4–37.8	4.10	10.15	-	3
		C7	*qIPRLLB-C7*	25.1	23.0–25.9	4.28	10.51	+	1, 2,3
		C8	*qIPRLLB-C8*	83.0	82.9–87	6.74	18.00	-	1, 2
	SDW	A3	*qSDWLB-A3a*	86.5	78.5–90.0	12.66	40.22	+	1, 2, 3
		A3	*qSDWLB-A3b*	103.8	102.6–107.9	7.10	14.57	-	1
		A4	*qSDWLB-A4*	85.3	74.7–85.7	9.19	20.65	+	1, 3
		A5	*qSDWLB-A5*	78.8	77.3–80.2	6.36	13.13	+	2
		C3	*qSDWLB-C3*	43.9	36.2–45.8	4.69	13.60	-	2, 3
		C7	*qSDWLB-C7*	108.0	100.5–109.3	5.75	17.70	+	3
	RDW	A3	*qRDWLB-A3a*	86.5	85.0–90.1	15.48	43.93	+	1, 2
		A3	*qRDWLB-A3b*	103.6	102.2–107.9	4.96	8.76	-	1
		A4	*qRDWLB-A4*	85.3	81.6–87.7	8.30	16.96	+	1, 3
		A5	*qRDWLB-A5*	78.8	75.3–80.8	3.33	8.38	+	2
		C3	*qRDWLB-C3*	41.9	39.9–48.2	4.52	14.75	-	3
		C4	*qRDWLB-C4a*	35.4	35.0–35.5	4.06	6.14	-	2
		C4	*qRDWLB-C4b*	94.9	93.3–96.3	5.15	17.25	+	3
	SBA	A3	*qSBALB-A3*	86.5	79.8–90.3	4.12	11.56	+	2, 3
		A4	*qSBALB-A4*	85.3	74.7–85.7	7.28	24.03	+	3
		A5	*qSBALB-A5*	77.8	75.9–80.2	4.61	11.87	+	2
	RBA	A3	*qRBALB-A3*	86.5	85.8–90.2	6.59	20.34	+	2
		A4	*qRBALB-A4*	85.3	72.3–85.7	8.21	13.76	+	2, 3
		A5	*qRBALB-A5*	128.4	124.5–133.4	5.93	21.03	-	3
		A6	*qRBALB-A6*	0.0	0.0–5.1	4.59	12.84	+	2
		A7	*qRBALB-A7*	62.4	59.2–64.8	4.52	12.65	-	2
—	BEC	A3	*qBEC-A3a*	86.5	83.0–90.2	9.09	30.79	+	1, 2, 3
		A3	*qBEC-A3b*	103.6	101.3–103.8	5.53	16.09	-	1, 2
		A4	*qBEC-A4*	85.3	74.7–85.7	4.26	13.85	+	1, 3
		C8	*qBEC-C8*	74.8	73.4–81.7	5.87	17.41	-	1

HB, high B; LB, low B; IPRL, increment of primary root length; SDW, shoot dry weight; RDW, root dry weight; SBA, shoot B accumulation; RBA, root B accumulation; BEC, B efficiency coefficient

*, Linkage group.

† , The peak position is denoted by the number in parentheses.

‡ , The 2-LOD confidence interval of QTL in centimorgans (cM).

§ , LOD score as calculated by WinQTLCart 2.5.

¶ , Phenotypic variation explained by each identified QTL.

# , Additive effect. Positive and negative effects are associated with contribution from Qingyou 10 and Westar 10, respectively.

ξ , Numbers indicate the trial in which the QTLs were detected.

### QTL for B uptake

In total, 12 QTLs associated with B uptake were identified, 4 detected at HB and 8 at LB. All QTLs for B uptake could explain more than 10% of the phenotypic variation, ranging from11.56–24.03% individually. Favorable alleles for increasing B uptake were contributed by Qingyou 10 at 8 loci and by Westar 10 at 4 loci (Table 4).

Five QTLs related to SBA were identified, 2 at HB and 3 at LB. Two QTLs controlling RBA at HB and five at LB were also found. Under LB, the intervals of *qSBALB-A3* and *qRBALB-A3* overlapped on A3. Similarly, *qSBALB-A4b* and *qRBALB-A4* were co-located on A4. The QTLs *qSBALB-A3* and *qRBALB-A4* were consistently identified in two trials.

### QTL for BEC

Four QTLs were observed for BEC, two of which were located on A3 and the others on A4 and C8. Favorable alleles for increasing BEC were contributed by Qingyou 10 at two loci and by Westar 10 at two loci. The major QTL for BEC, *qBEC-A3a*, explained 30.79% of the phenotypic variation and was detected in all three trials. Three other minor QTLs for BEC accounted for 16.09, 13.85 and 17.41% of the phenotypic variation, respectively (Table 4).

### Co-location analysis of QTLs for BEC, plant growth and B uptake traits

**Figure 2:**
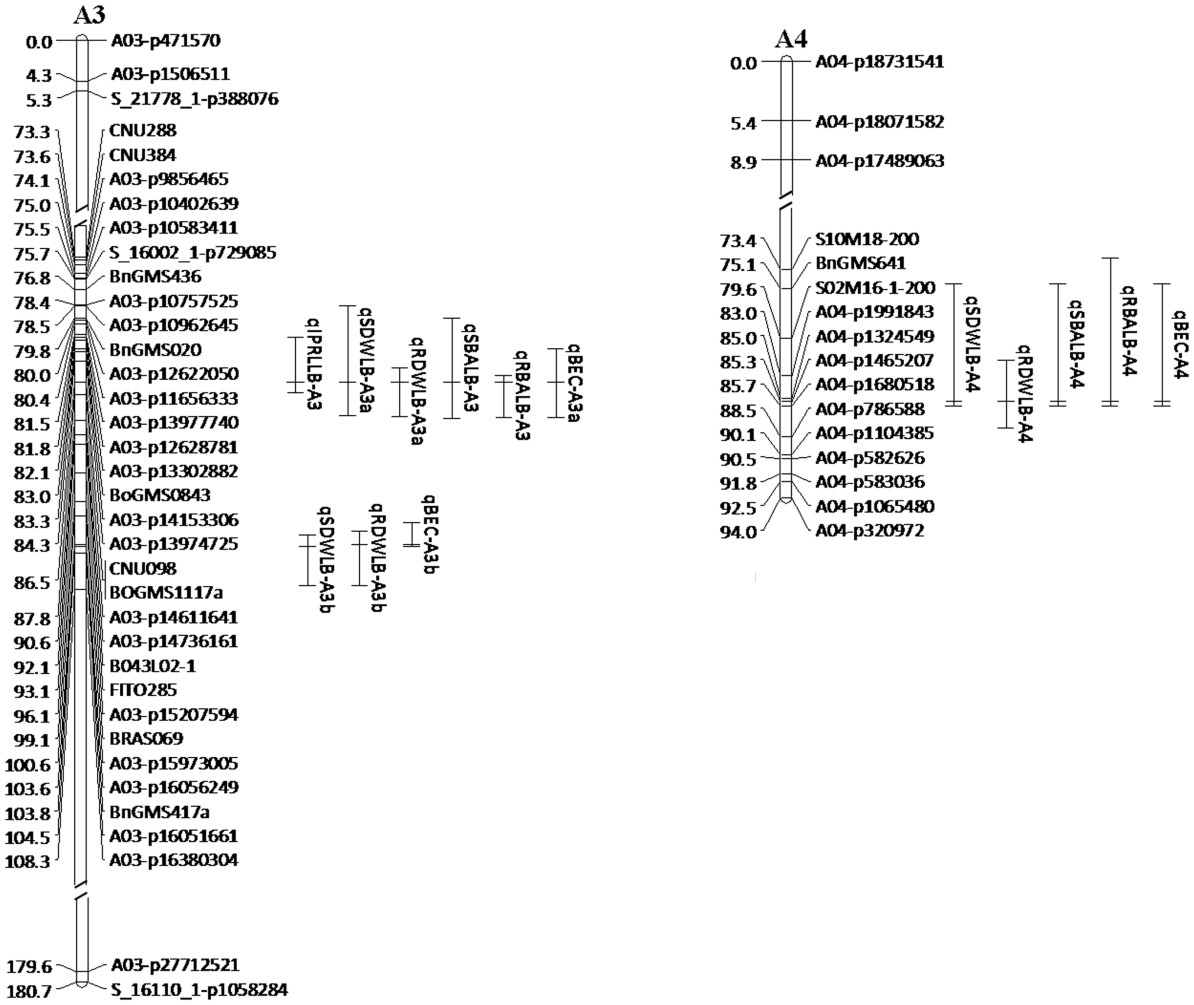
Co-location of QTLs for the B efficiency coefficient (BEC), plant growth and B uptake traits in the QW DH population.

Further QTL linkage analysis found that three of four QTLs for BEC were co-located with the QTLs for plant growth and B uptake traits at LB (Fig. 2). The major QTL for BEC, *qBEC-A3a*, co-located with the major QTLs for IPRL, SDW, RDW, RBA and a minor QTL for SBA at the peak position of 86.5 cM on A3, constituting a QTL cluster, A3a. Another QTL for BEC on A3, *qBEC-A3b*, overlapped with the QTLs for SDW and RDW, forming another QTL cluster, A3b. Interestingly, favorable alleles of all QTLs in cluster A3a were derived from the B-efficient parent Qingyou 10 and in cluster A3b from the B-inefficient parent Westar 10. Another QTL cluster associated with BEC, SDW, RDW, SBA, and RBA was found at the peak position of 85.3 cM on A4. Favorable alleles of all QTLs in this cluster were derived from the B-efficient parent Qingyou 10.

### Validation of effect of *qBEC-A3a*

**Figure 3:**
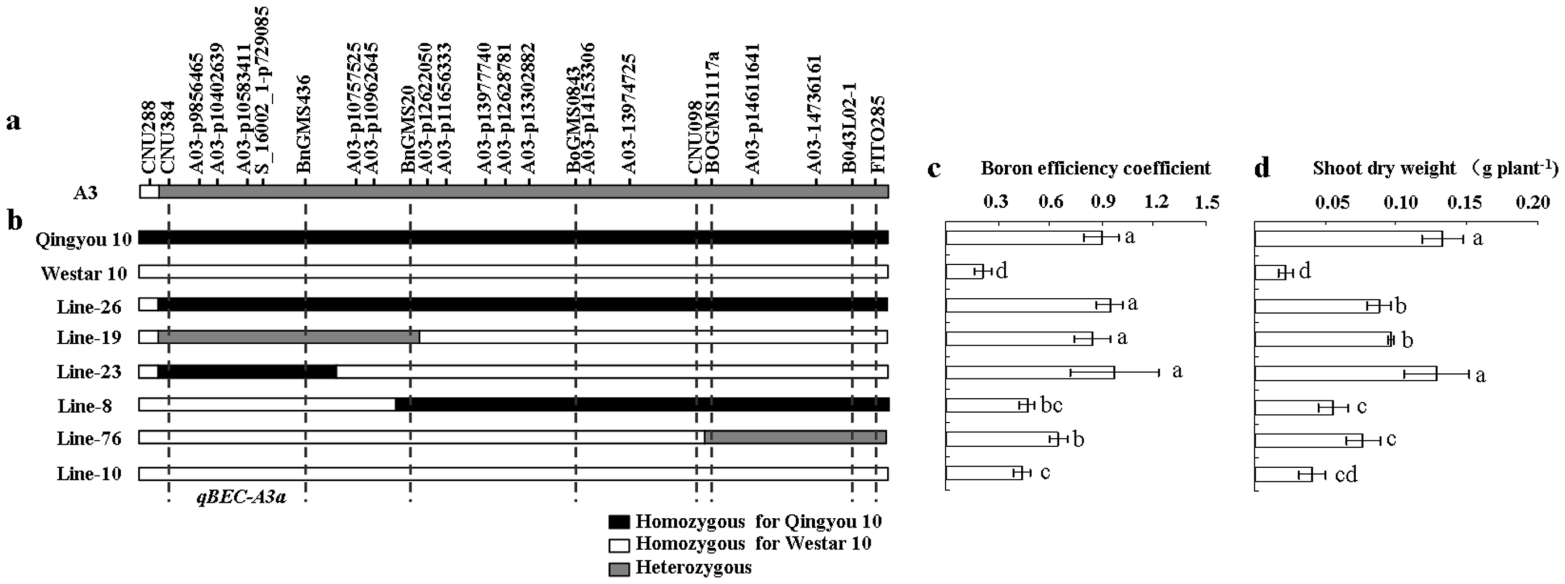
Schematic chromosomal fragments of substitution lines. a The region of *qBEC-A3a* in the A3 linkage group based on the QW DH population. b Substitution lines screened from the BC_4_F_2_ population using eight SSR markers. The black bar represents the Qingyou 10 allele, the white bar represents the Westar 10 allele, and the grey bar represents the heterozygous genotype. c Boron efficiency coefficient (BEC) of each substitution line from the BC_4_F_2;3_ family. d Shoot dry weight (SDW) values at low B for each substitution line from the BC_4_F_2;3_ family.

To validate the effect of *qBEC-A3a* on B efficiency, one plant was screened from the BC_4_F_1_ population and determined to be heterozygous in the genomic region between SSR markers CNU384 and FITO285, in the confidence interval of *qBEC-A3a* (Fig. 3a), but homozygous for the other QTLs loci with the recurrent parent Westar 10. Six substitution lines, line-26, line-23, line-19, line-8, line-76 and line-10, were screened from a BC_4_F_2_ population developed by selfing of the genotyped BC_4_F_1_ plant (Fig. 3b). Among the six substitution lines, lines (line-26, line-23 and line-19) with a homozygous or heterozygous genomic region between CUN384 and BnGMS436 from Qingyou 10 showed significantly higher BEC and SDW at LB than those lines (line-8, line-76 and line-10) with the homozygous allele from Westar 10 (Fig. 3c, d). This suggests that *qBEC-A3a* had a pleiotropic effect on both BEC and SDW at LB and can be narrowed to the interval of CNU384 and BnGMS436.

## Discussion

### Construction of a high-density *B. napus* linkage map

Most previous studies on B efficiency in *B. napus* were carried out with lower-density genetic maps [26]–[28]. In this study, a high-density genetic map, covering a total length of 2,139.5 cM, was constructed using the QW DH population containing 70 lines (Table 1). To ensure the accuracy of the map in this small population, we first constructed a framework map using SSR markers according to the order of SSR markers in a previous published study [27], and then SNP-bins and AFLP markers were added to the genetic map. Many markers had skewed segregation ratios in the QW DH population (File S1). Skewed segregation ratios have also been observed in other DH populations for many plant species [47]–[48] and are potentially related to selection during microspore culture. Skewed ratios can easily lead to illegitimate joining of linkage groups. We manually removed illegitimate markers by referring to the known locations of most SNPs [49]. Finally, the high-density map contained 936 SNP-bins, 343 SSR and 119 AFLP markers (Table 1).

### Phenotypic variation across different B levels

Having suitable parental genotypes is critical for identifying important QTLs. In the early studies, the QTLs associated with B efficiency in *B. napus* were detected using F_2_ or DH populations derived from a cross between the B-efficient parent Qingyou 10 and the B-inefficient parent Bakow [26]–[28]. However, the B-inefficient parent Westar 10 used in the present study showed greater sensitivity to B deficiency than Bakow, as reported in a previous study [32]. In the present study, we found that the B-efficient line Qingyou 10 showed a significant advantage in plant growth and B uptake at LB and had a higher BEC than Westar 10 (Table 2).

Phenotypic investigation showed abundant variation for all traits in the QW DH population (Table 2, Fig. 1, File S2). Compared with HB, B deficiency resulted in a greater coefficient of variation (CV%) for the plant growth and B uptake traits (Table 2), suggesting that some B efficiency genes are involved in the phenotypic separation of the QW DH population in response to low B stress.

Generally, the variation in some individual traits in the population, such as PRL_0d_ and seed weight, might affect the phenotype of the QW DH population in response to B deficiency. In the present study, the potential correlation between PRL_0d_ and IPRL at LB was analyzed, but no significant correlation was found between two traits (File S3a), implying that PRL_0d_ had no significant effect on root elongation. Seed weight can indirectly reflect the amount of nutrition stored in the seed. In the present study, we found that seed weight had no significant effect on the SDW or RDW of the QW DH population at LB (File S3b). In fact, the symptoms of B deficiency appeared first in the younger leaves and the growing apex, implying that the nutrition in the seed is completely exhausted during germination and the very early stages of plant growth.

### QTLs detected for B-efficiency traits

Studies on the inheritance of B efficiency in *B. napus* have shown that it is a quantitative trait [50]. QTLs for B efficiency traits in *B. napus* have been reported using different populations [26]–[28]. In our study, a total of 52 QTLs were detected, including 36 associated with plant growth traits (IPRL, SDW and RDW), 12 for B uptake traits (SBA and RBA) and 4 conferring BEC (Table 4). Our study and the previously published works [26]–[28] had no QTLs in common. We presume that this may be because the traits were investigated at the maturity stage in the previous studies, while the traits in our study were investigated at the seedling stage. In addition, the effect of population size on power of QTL detection as well as on accuracy and precision of QTL estimates was large [51]. QTL with large effects were detected even in small population, however, QTL with small effects were detected only by increasing population size [52]. So it is possible that the lack of correspondence of QTLs in this study and previous studies could relate to the relatively small population size used in this study. However, we believe that QTLs detected in two or more trials and with high heritability estimates (Tables 2 and 4) in this present study are highly credible.

Due to the strong positive correlation between the tested traits across B levels (Table 3), it is not surprising that the QTLs for different traits overlapped with each other. The positive correlation and co-localization of QTLs among the tested traits suggests that these physiological processes are regulated at the same molecular level. In the current study, the QTLs for BEC were observed to overlap with the QTLs for plant growth and B uptake traits on A3 and A4 linkage groups (Fig. 2). The major QTL for BEC, *qBEC-A3a*, was located at the peak position of 86.5 cM on A3, which also simultaneously confers IPRL, SDW, RDW, SBA and RBA at LB. Furthermore, the favorable allele from Qingyou 10 located between the marker CNU384 and BnGMS436 increased the BEC and SDW at LB of substitution line (Fig. 3c, d). This finding suggests that the QTL locus on A3a is slightly associated with B efficiency in *B. napus*. To our knowledge, *qBEC-A3a* is a novel major QTL for B efficiency, distinct from the major gene *BE1* detected for B efficiency and seed yield in the previous study [26]. The large effect and stability of *qBEC-A3a* make it suitable for fine mapping and map-based cloning to uncover the molecular mechanisms of B deficiency tolerance in *B. napus* at the seedling stage.

Six B transporter genes (*BnBOR1*s) homologous to *Arabidopsis AtBOR1* were cloned in *B. napus* [19], which presented divergent expression pattern in *B. napus*. *BnBOR1;3a* and *BnBOR1;3c* showed a ubiquitous expression in all of the investigated tissues, whereas the other four genes showed similar tissue-specific expression profile. Among these six *BnBOR1*s, the expression of *BnBOR1;1c* and *BnBOR1;2a* were obviously induced by B deficiency. Further, Yang et al [32] used two B-efficient and two B-inefficient *B. napus* cultivars to compare the expression of *BnBOR1*s by RT-PCR, and found that there was no obvious difference in the expression of *BnBOR1*s between B-efficient and B-inefficient cultivars in low B or normal B conditions. With the recent publication and release of *B. napus* genome sequence (http://www.genoscope.cns.fr/colza-ggb/cgi-bin/gbrowse_syn/colza/) [53], the six *BnBOR1*s previously identified were physically mapped on chromosome A3, A4, A5, C3 and C4, respectively. Among them, *BnBOR1;3a* was located in the region of *qBEC-A3a*. However, the expression of *BnBOR1;3a* showed no obvious difference between Qingyou 10 and Westar 10 under low B condition in the previous study [32]. Taken together, it can be concluded that B efficiency in *qBEC-A3a* locus of *B. napus* may be regulated by an unknown gene. Accordingly, fine mapping and screening of the candidate gene for *qBEC-A3a* based on map-based cloning and bioinformation should be proceeded in the near future, which will lay a solid foundation for uncovering the genetic and molecular mechanism on the tolerance of *B. napus* to B deficiency.

## Supporting Information

File S1 **Full list of SNP-bin, SSR and AFLP markers, map positions, allele ratios and chi-square P-values for each marker in the high-density genetic map of the QW DH population.**
(XLS)

File S2 **Means of tested traits for each lines in the QW DH population derived from a cross between Qingyou 10 and Westar 10 under high and low B.**
(XLSX)

File S3 **Relations between original primary root length (PRL_0d_) and increment of primary root length (IPRL) (a), seed weight, shoot dry weight (SDW), root dry weight (RDW) (b) at low B.**
(TIF)
